# Recognition and management of persistent chylomicronemia: A Joint Expert Clinical Consensus by the National Lipid Association and the American Society for Preventive Cardiology

**DOI:** 10.1016/j.jacl.2025.03.012

**Published:** 2025-03-28

**Authors:** Seyedmohammad Saadatagah, Miriam Larouche, Mohammadreza Naderian, Vijay Nambi, Diane Brisson, Iftikhar J. Kullo, P. Barton Duell, Erin D. Michos, Michael D. Shapiro, Gerald F. Watts, Daniel Gaudet, Christie M. Ballantyne

**Affiliations:** Department of Medicine, Baylor College of Medicine, Houston, TX, USA; Center for Translational Research on Inflammatory Diseases, Baylor College of Medicine, Houston, TX, USA; Department of Medicine, Université de Montréal, Montreal, Canada; ECOGENE-21, Chicoutimi, Canada; Department of Cardiovascular Medicine, Mayo Clinic, Rochester, MN, USA; Department of Medicine, Baylor College of Medicine, Houston, TX, USA; Michael E. DeBakey Veterans Affairs Hospital, Houston, TX, USA; Department of Medicine, Université de Montréal, Montreal, Canada; ECOGENE-21, Chicoutimi, Canada; Department of Cardiovascular Medicine, Mayo Clinic, Rochester, MN, USA; Gonda Vascular Center, Mayo Clinic, Rochester, MN, USA; Knight Cardiovascular Institute and Division of Endocrinology, Diabetes, and Clinical Nutrition, Oregon Health and Science University, Portland, OR, USA; Division of Cardiology, Johns Hopkins University School of Medicine, Baltimore, MD, USA; Section of Cardiovascular Medicine, Center for Prevention of Cardiovascular Disease, Wake Forest University School of Medicine, Winston-Salem, NC, USA; Medical School, University of Western Australia, Perth, Australia; Cardiometabolic Service, Departments of Cardiology and Internal Medicine, Royal Perth Hospital, Perth, Western Australia, Australia; Department of Medicine, Université de Montréal, Montreal, Canada; ECOGENE-21, Chicoutimi, Canada; Department of Medicine, Baylor College of Medicine, Houston, TX, USA

**Keywords:** Pancreatitis, ApoC-III inhibitors, Familial chylomicronemia syndrome, Multifactorial chylomicronemia syndrome, Persistent chylomicronemia

## Abstract

Extreme hypertriglyceridemia, defined as triglyceride (TG) levels ≥1000 mg/dL, is almost always indicative of chylomicronemia. The current diagnostic approach categorizes individuals with chylomicronemia into familial chylomicronemia syndrome (FCS; prevalence 1–10 per million), caused by the biallelic combination of pathogenic variants that impair the lipolytic action of lipoprotein lipase (LPL), or multifactorial chylomicronemia syndrome (MCS, 1 in 500). A pragmatic framework should emphasize the severity of the phenotype and the risk of complications. Therefore, we endorse the term “persistent chylomicronemia (PC)” defined as TG ≥1000 mg/dL in more than half of the measurements to encompass patients with the highest risk for pancreatitis, regardless of their genetic predisposition. We suggest classification of PC into 4 subtypes: (1) genetic FCS, (2) clinical FCS, (3) PC with “alarm” features, and (4) PC without alarm features. Although patients with FCS most likely have PC, the vast majority with PC do not have genetic FCS. Proposed alarm features are: (a) history of recurrent TG-induced acute pancreatitis, (b) recurrent hospitalizations for severe abdominal pain without another identified cause, (c) childhood pancreatitis, (d) family history of TG-induced pancreatitis, and/or (e) postheparin LPL activity <20% of normal value. Alarm features constitute the strongest risk factors for future acute pancreatitis risk. Patients with PC and alarm features have very high risk of pancreatitis, comparable to that in patients with FCS. Effective, innovative treatments for PC, like apolipoprotein C-III inhibitors, have been developed. Combined with lifestyle modifications, these agents markedly lower TG levels and risk of pancreatitis in the very-high-risk groups, irrespective of the monogenic etiology. Pragmatic definitions, education, and focus on patients with PC, specifically those with alarm features, could help mitigate the risk of acute pancreatitis and other complications.

## Triglyceride-rich lipoproteins: Chylomicrons and very-low-density lipoproteins

**Figure 1:**
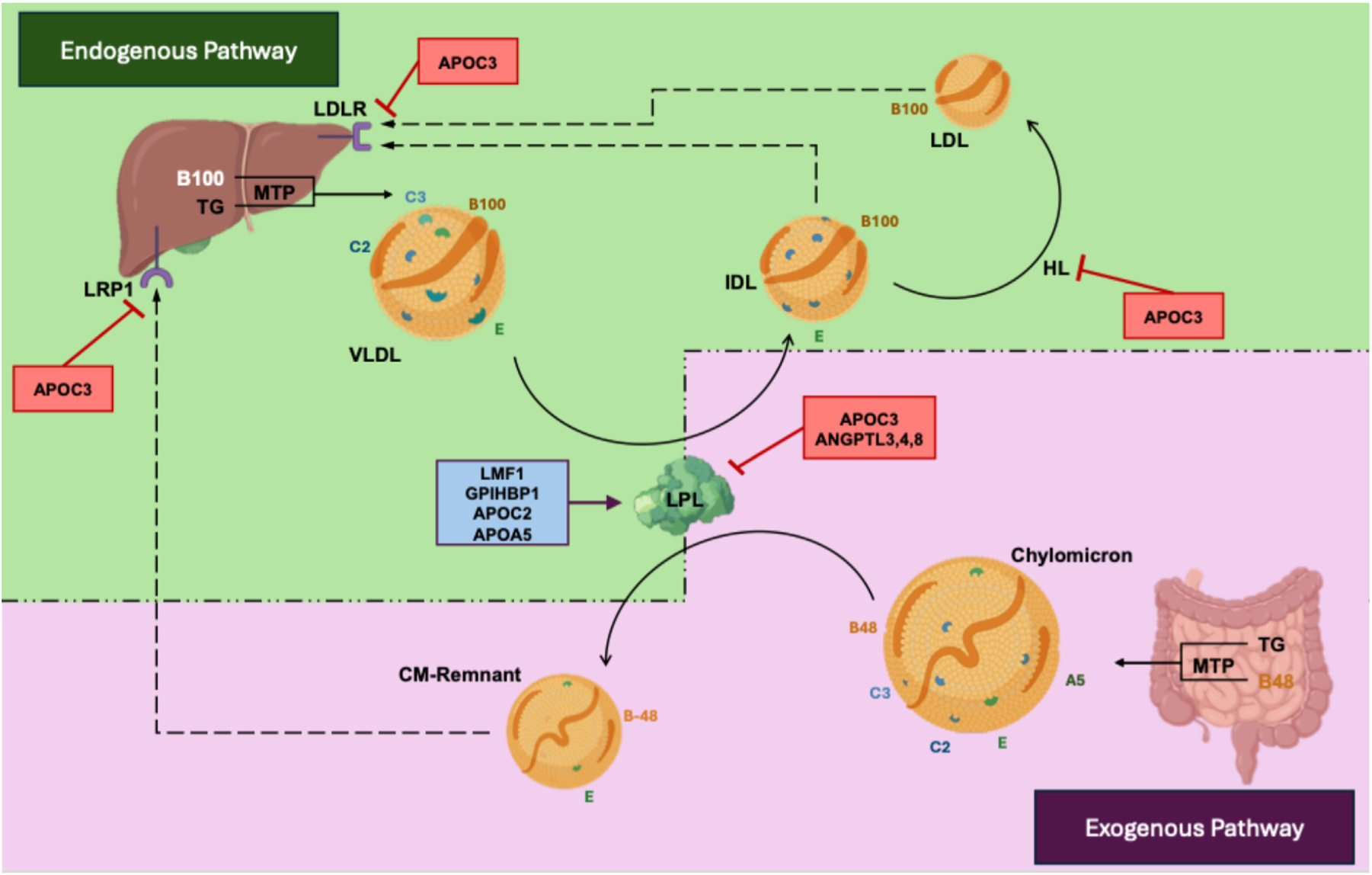
Triglyceride-rich lipoprotein metabolism. Abbreviations: A5, apolipoprotein A-V; ANGPTL, angiopoietin-like protein; B48, apolipoprotein B-48; B100, apolipoprotein B-100; C2, apolipoprotein C-II; C3, apolipoprotein C-III; CM-remnant, chylomicron remnant; DGAT, diacylglycerol acyltransferase; E, apolipoprotein E; FA, fatty acid; GPIHBP1, glycosylphosphatidylinositol-anchored high-density lipoprotein–binding protein 1; HL, hepatic lipase; IDL, intermediate-density lipoprotein; LDL, low-density lipoprotein; LDLR, low-density lipoprotein receptor; LMF1, lipase maturation factor 1; LRP1, LDL receptor–related protein 1; MTP, microsomal triglyceride transfer protein; PCSK9, proprotein convertase subtilisin/kexin type 9; TG, triglyceride; VLDL, very-low-density lipoprotein. Created in BioRender. Saadatagah, S. (2025) https://BioRender.com/f90b640.

The metabolism of TRL involves a complex interplay of diverse, key regulatory proteins, cofactors, and enzymes. In the intestine (exogenous pathway), chylomicrons are formed from absorbed dietary fat with apolipoprotein B-48 (apoB-48) as the main structural protein, while in the liver (endogenous pathway), very-low-density lipoproteins (VLDL) are assembled from hepatic triglycerides (TG) with apolipoprotein B-100 (apoB-100).^1^ Microsomal triglyceride transfer protein (MTP) plays a crucial role in both processes. After secretion into the circulation, these TRL rapidly undergo lipolytic depletion of TG primarily mediated by lipoprotein lipase (LPL), a key enzyme in large TRL metabolism, modulated by a series of regulatory proteins and cofactors^1,2^ (Fig 1). Four proteins are required for LPL activity: (1) lipase maturation factor 1 (LMF1) ensures proper folding and secretion of LPL, (2) glycosylphosphatidylinositol-anchored high-density lipoprotein binding protein-1 (GPI-HBP1) is crucial for translocating LPL across the capillary endothelium and anchoring it on the luminal surface, (3) apolipoprotein C-II (apoC-II) acts as an essential activator of LPL, and (4) apolipoprotein A-V (apoA-V) serves as a stabilizing cofactor. These 5 proteins are hereafter called LPL machinery. On the other hand, apolipoprotein C-III (apoC-III) and angiopoietin-like proteins (ANGPTLs), specifically ANGPTL3, ANGPTL4, and ANGPTL8, inhibit LPL activity, thereby reducing lipolysis.^2^ Rare loss-of-function variants affecting genes encoding LPL, apoA-V, apoC-II, LMF1, and GPIHBP1 are pathogenic and can cause chylomicronemia, whereas loss-of-function variants in genes encoding MTP, apoC-III, and ANGPTL3 can result in low TG levels.^1,2^

## Hypertriglyceridemia spectrum and identification of patients with chylomicronemia

**Figure 2:**
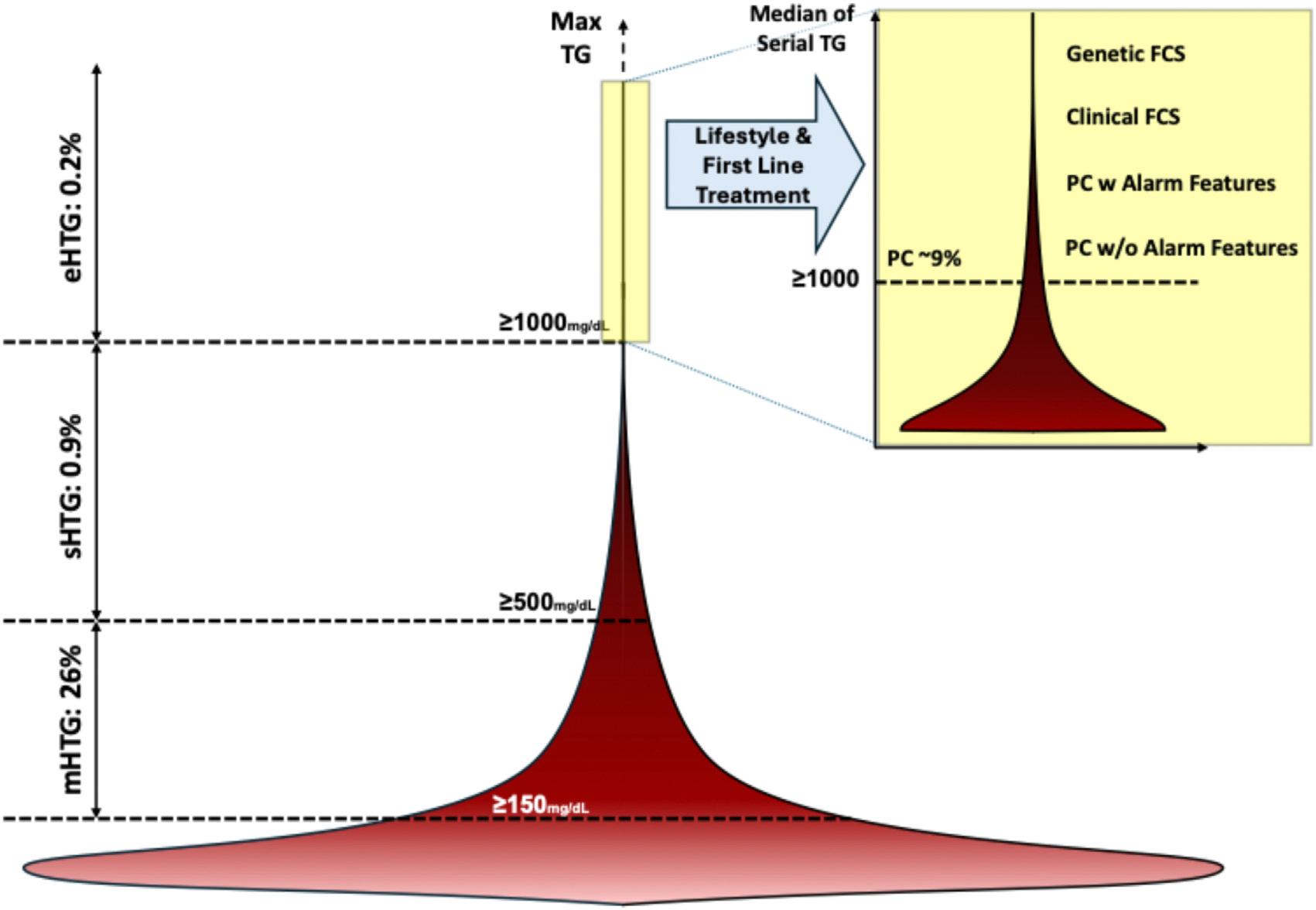
The spectrum of triglyceride elevation. The right-skewed bell represents the distribution of fasting triglycerides based on the National Health and Nutrition Examination Survey (NHANES) data. The box on the top right magnifies the distribution of median TG in those with eHTG. TG ≥1000 mg/dL in more than half of the TG measurements (ie, median TG ≥1000 mg/dL) is defined as PC. Abbreviations: eHTG, extreme hypertriglyceridemia; FCS, familial chylomicronemia syndrome; mHTG, mild–moderate hypertriglyceridemia; PC, persistent chylomicronemia; sHTG, severe hypertriglyceridemia; TG, triglycerides. Figure adapted from Saadatagah S, et al. *J Clin Lipidol.* In press.^11.^

The distribution of plasma TG in the population is highly skewed to the right (Fig 2).^3^ Normal fasting TG levels are typically defined as <150 mg/dL, mild–moderate hyper triglyceridemia (mHTG) as TG levels 150 to 499 mg/dL, severe hypertriglyceridemia (sHTG) as TG levels ≥500 mg/dL, and extreme hypertriglyceridemia (eHTG) as TG levels ≥1000 mg/dL.^4^ The presence of chylomicrons in the blood-stream is normal in the postprandial state but not after an overnight (10–12 hour) fast.^5^ In a clinical setting, eHTG is commonly considered as an operational definition of chylomicronemia, although chylomicrons may contribute to the pool of circulating TRL in some patients with sHTG.^6^

Fasting chylomicronemia is estimated to affect between 0.1% and 0.2% of people in European countries.^7,8^ The prevalence could be higher in certain communities in French Canada, the Middle East, South Africa, and Southern Asia because of founder effects, consanguinity, or other factors affecting glycemia and metabolic syndrome at the population level.^9,10^ A recent study from the United States showed that among adults ≥20 years old who had participated in the National Health and Nutrition Examination Survey (NHANES) from 1999 to 2018 and had fasting TG measured, 72.7% had TG <150 mg/dL, 26.2% had TG 150 to 499 mg/dL, 0.9% had TG 500 to 999 mg/dL, and 0.20%, equating to approximately 1 in 500 American adults, had eHTG, defined as TG ≥1000 mg/dL, reflecting chylomicronemia.^11^

## Current approach to classifying chylomicronemia: Familial chylomicronemia and multifactorial chylomicronemia syndromes

Over the last century, several different clinical labels have been used to describe eHTG, which are reviewed in detail elsewhere.^12^ The current classification of chylomicronemia differentiates genetically confirmed FCS, caused by biallelic pathogenic variants in genes critical for LPL function, from MCS, which encompasses all other forms arising from a combination of genetic predispositions, environmental influences, and secondary factors.^13,14^ Table 1 outlines the key differences between FCS and MCS.

FCS, a rare disease affecting approximately 1 to 10 individuals per 1,000,000,^15,16^ is an autosomal recessive disease caused by the biallelic combination of pathogenic variants that impair the lipolytic action of LPL, either defects in *LPL* or in the genes encoding cofactors and proteins required for LPL processing and function (ie, *LMF1, GPIHBP1, APOC2,* and *APOA5*). Disruption of LPL function severely impairs the lipolysis and subsequent clearance of chylomicron and VLDL particles from circulation, leading to extremely elevated TG levels and chylomicronemia. Individuals with FCS often present with symptoms in childhood or adolescence, and have high risk for recurrent acute pancreatitis due to severe refractory chylomicronemia.^17–19^ Nongenetic factors such as insulin resistance, impaired glucose tolerance, diabetes, obesity (particularly visceral adiposity), certain medications, ethanol consumption, and poor dietary habits significantly exacerbate chylomicronemia severity. However, in patients with FCS, strict control of these modifiable factors rarely normalizes TG levels because of the profound underlying genetic impairment of LPL function.^2,16,20^ Individuals with FCS typically have chylomicronemia and very low levels of low-density lipoprotein cholesterol (LDL-C) and do not develop premature cardiovascular disease. Some FCS patients with additional risk factors accumulate atherogenic remnant particles in plasma, which increase the risk of atherosclerotic cardiovascular disease (ASCVD) events.^21,22^ A small subgroup of individuals who do not have biallelic pathogenic variants in the LPL machinery exhibit all the clinical phenotypic characteristics of FCS.^3,16^ These individuals are sometimes classified as having clinical FCS.^12,19^

Compared with FCS, MCS is at least 3 orders of magnitude more common,^12^ arising from a combination of genetic predisposition and environmental influences. Although TG levels are elevated in MCS, they are generally lower and more labile than in FCS. MCS may result from a combination of rare loss-of-function variants in genes involved in TG metabolism, including heterozygosity in the canonical genes involved in the LPL machinery, and secondary factors such as visceral adiposity, insulin resistance, impaired glucose tolerance, diabetes, consumption of ethanol, suboptimal dietary composition, and certain medications (Table 2).^15,24,26^ These factors can contribute to hypertriglyceridemia by increasing production and/or decreasing clearance of TRL; of note, large VLDL and chylomicrons share a common catabolic pathway via LPL.^20,27^ MCS can present at any age, though it typically manifests later than FCS, and is often associated with lower risk for acute pancreatitis than FCS, but MCS sometimes is associated with extreme risk for pancreatitis. However, because of potentially broader perturbations in lipid metabolism, involving the accumulation of VLDL, intermediate-density lipoproteins (IDL), and small dense LDL, as well as greater prevalence of ASCVD risk factors such as obesity, metabolic syndrome, and diabetes, patients with MCS generally have far higher risk of ASCVD than do patients with FCS.^20,25,27^ In the absence of genetic testing, clinical diagnosis scoring systems, including the North American FCS Score Calculator and the European FCS Score, have been developed to help differentiate FCS from MCS.^18,19,28^ These scoring systems use a combination of clinical criteria, lipid profile data, age at onset, family history, response to traditional TG-lowering agents, and presenting symptoms to assess the likelihood of a patient having little or no LPL function, which could be genetically confirmed as FCS, if desired.^29^ Two scores were tested and correlated fairly well with postheparin LPL activity.^29^ However, these scoring systems are designed to distinguish FCS cases from MCS, not the clinical outcome; whether they will improve the care of people with chylomicronemia needs to be established.

## Risks associated with chylomicronemia

Chylomicronemia is associated with eruptive xanthomas, lipemia retinalis, hepatosplenomegaly, metabolic dysfunction–associated steatotic liver disease (MASLD), abdominal pain, recurrent acute pancreatitis (with onset as early as infancy), and generalized symptoms such as fatigue, dyspnea, and cognitive impairment (“brain fog”).^12,25,35^ Severe TG elevation is the third most common cause of acute pancreatitis (after cholelithiasis and excess ethanol consumption), which can lead to temporary or permanent organ dysfunction, pancreatic necrosis, and death in 1% to 5% of cases.^36^ Most knowledge regarding pathophysiological mechanisms of acute pancreatitis is derived from animal studies.^37^ The 2 main mechanistic theories about how extremely elevated TG initiates and/or aggravates acute pancreatitis are (1) increased levels of free fatty acids (FFAs) in the pancreas resulting from hydrolysis of chylomicron TG by pancreatic lipases leading to activation of an inflammatory response and (2) increased blood viscosity due to high levels of chylomicrons resulting in microcirculatory abnormalities.^38–41^ Chylomicronemia-induced acute pancreatitis is associated with worse clinical outcomes, including longer hospital stays, pancreatic necrosis, persistent organ failure, and higher mortality rates, compared with other causes of acute pancreatitis.^42,43^ Mendelian randomization data suggest a causal role of TG elevation in acute pancreatitis, with risk proportional to TG levels.^37,44^ After an initial episode of acute pancreatitis, recurrent acute pancreatitis events may occur at lower TG thresholds, and chronic pancreatitis may also occur.^45,46^ When eHTG includes the accumulation of lipoproteins in both the exogenous (ie, chylomicrons and their remnants) and endogenous (ie, VLDL and IDL) pathways, such as in MCS, affected patients have a greater risk for ASCVD.^27^

## Rationale for a new approach to the classification of chylomicronemia

The current approach to categorizing patients with chylomicronemia into FCS and MCS is historically based but has several limitations.^12^ First, only about 0.1% to 1% of patients with chylomicronemia have FCS; although the rate of pancreatitis is higher in FCS patients, the vast majority of individuals with pancreatitis due to chylomicronemia have MCS, related to its dramatically greater prevalence.^19^ Similar to other complex traits, the genetics of chylomicronemia may be better characterized by combining polygenic and monogenic models. In this framework, a high polygenic TG score could exert a TG-raising effect equal to or greater than that of a monogenic variant.^47,48^ This is evident from the understanding that more than one-third of patients with clinical FCS do not carry known biallelic combinations of FCS-causing pathogenic variants.^19^ There is also large variability in TG levels among patients carrying the same FCS variants, emphasizing the importance of other genetic and nongenetic factors in modulating the severity of chylomicronemia.^49^ Additionally, nongenetic causes, such as blocking autoantibodies directed against LPL^50,51^ or GPIHBP1,^52^ and complex epigenetic modifications^53^ can cause the FCS phenotype. In addition, genetic testing is not routinely performed in clinical practice and is not always accessible in many parts of the world, particularly in middle- and lower-income countries.^54^ Reliance on genetic testing to differentiate between FCS and MCS may exacerbate existing healthcare disparities. It has been demonstrated that individuals from minority groups and economically disadvantaged backgrounds are less likely to undergo genetic testing, which could limit their access to newer medications as a result of underdiagnosis.^55,56^ Moreover, the term “familial” is misleading; patients with FCS usually do not have a family history of chylomicronemia because it is an autosomal recessive disorder, whereas patients with MCS often have a family history of elevated TG.^2^

Given the well-established causal relationship between chylomicronemia and pancreatitis, which is similar to the causal relationship between LDL-C and ASCVD, we can draw an analogy to support the rationale for an updated clinical approach to the classification of chylomicronemia. Familial hypercholesterolemia (FH) is recognized as a monogenic cause of markedly elevated LDL-C, primarily associated with mutations in *LDLR, APOB, PCSK9,* or *LDLRAP1*. However, most individuals (>90%–95%) with severe hypercholesterolemia (ie, LDL-C ≥190 mg/dL) do not have a positive genetic test, and LDL-C levels vary markedly among patients with the same gene variant.^47,48^ Although a genetic diagnosis of FH is valuable for purposes such as cascade family screening, management is tailored based on the extent of LDL-C elevation and risk of complications, particularly ASCVD.^57,58^

By analogy, a similar paradigm can be applied to the classification and management of chylomicronemia, with a focus on the magnitude and duration of TG elevation and the risk of complications, particularly pancreatitis. Focusing exclusively on the rare subgroup of genetically confirmed FCS patients for novel therapies inadequately addresses the broader clinical challenge among the 1000-fold more prevalent group of patients with MCS, among whom the majority of cases of acute pancreatitis and other morbidities of chylomicronemia occur. A broader strategy emphasizing risk reduction and effective management, regardless of the genetic basis, is essential to meet the needs of all high-risk patients with chylomicronemia. Therefore, we propose the term “persistent chylomicronemia (PC)” to encompass patients with the highest risk for pancreatitis, regardless of their genetic predisposition, and recommend treatment strategies based on the severity of TG elevation and the risk of complications, particularly pancreatitis.

## Temporal patterns of triglyceride: Spectrum of chylomicronemia burden

The lability of plasma TG levels makes it challenging for any classification based on temporal patterns of TG levels.^11,59^ Chylomicronemia severity and frequency could be considered across a spectrum ranging from a single occurrence in a lifetime to persisting in every measurement. Infrequent and sporadic episodes of chylomicronemia are usually caused by secondary factors that occur episodically, such as high ethanol intake or a temporary increase in dietary fat intake, in the context of a genetic predisposition. Whether TG levels normalize between these episodes of chylomicronemia depends on underlying metabolic conditions and aggravating factors affecting the metabolism of TRL. More frequent and persistent episodes of eHTG suggest a stronger genetic influence and/or more enduring uncontrolled secondary factors, such as uncontrolled diabetes, adverse dietary habits, or ethanol intake.^2,3,12–14^

It is suggested that the term “persistent” in PC should not be considered in its literal sense, as occasional decreases in TG below the chylomicronemia threshold of 1000 mg/dL do not exclude PC. For instance, the TG level in genetically confirmed FCS patients in the Balance trial ranged from 334 to 6898 mg/dL.^60^ Therefore, the appropriate cut point for defining PC is unclear. The classification for treatment could include an estimation of risk of complications, particularly pancreatitis, noting that such risk increases markedly when severe PC is present.^25^

In a recent study in 1,294,044 individuals from a large healthcare network across 3 US states (Minnesota, Florida, and Alabama), 5618 (0.43%) patients had at least 1 episode of chylomicronemia.^11^ The investigators aimed to define pancreatitis risk based on the proportion of TG levels measuring ≥1000 mg/dL out of the total number of TG measurements, using cutoffs of 50%, 75%, and 83%. Among individuals with chylomicronemia who had multiple TG measurements, 8.8%, 1.6%, and 0.7% met the respective cutoffs. In comparison, the overall occurrence of pancreatitis increased only slightly across these cutoffs, rising from 28% to 29% to 30%.^11^ To put the numbers in perspective, among 775,019 individuals with maximum TG <150 mg/dL, 1.3% had a diagnosis of acute pancreatitis, compared with 12.5% of individuals with non-PC (less than half of TG measurements ≥1000 mg/dL).^11^ Therefore, investigators defined PC as TG levels ≥1000 mg/dL in more than half of multiple measurements. Using this definition, only 8.8% of patients with chylomicronemia (1:5500 in the general US population) met this operational definition of PC. Younger age, Hispanic ethnicity, history of pancreatitis, and higher TG levels were predictors of PC.^11^

## Persistent chylomicronemia and its subtypes

**Figure 3:**
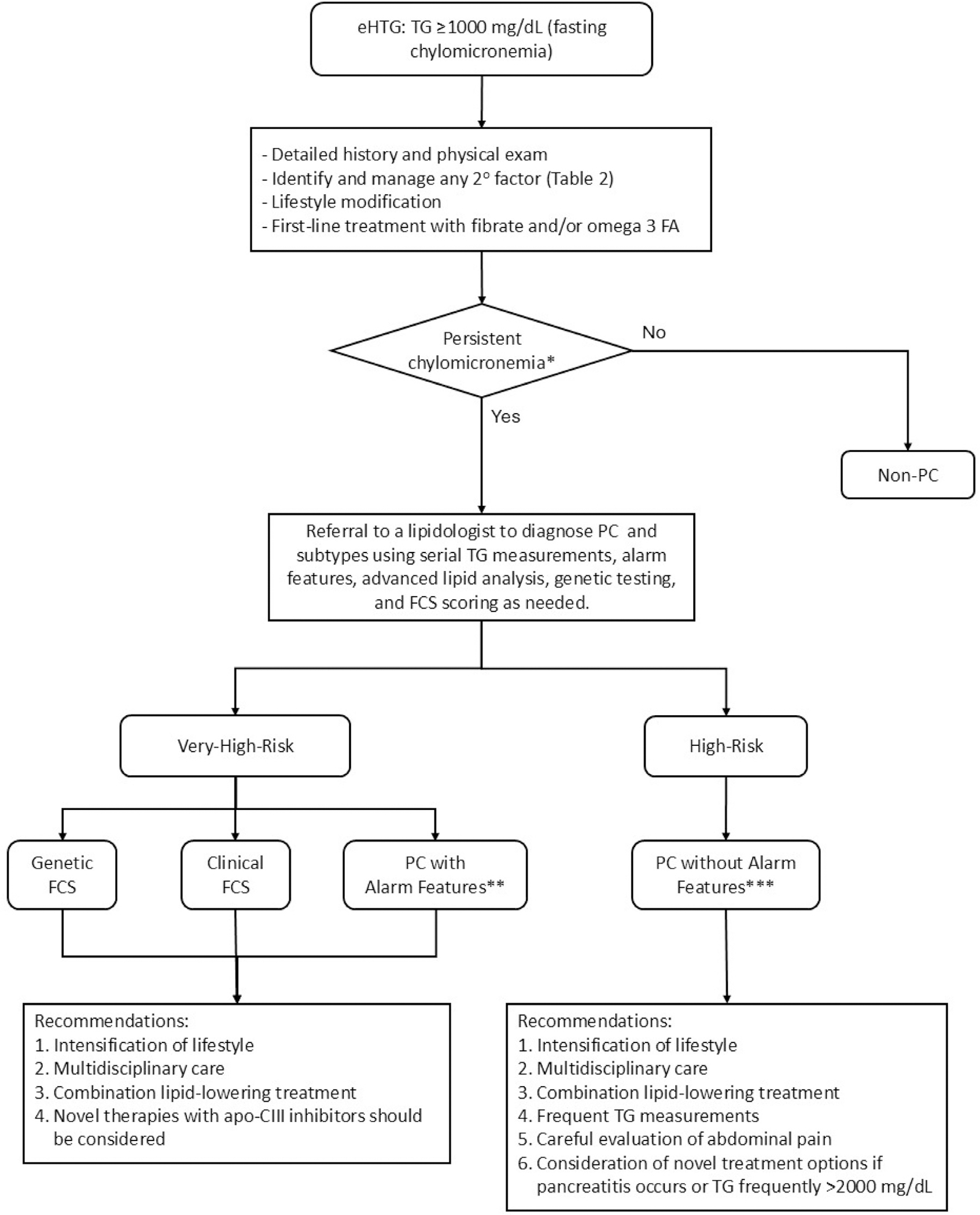
Proposed algorithm to diagnose and manage PC. Patients with genetic FCS, clinical FCS, and PC with alarm features have very high and similar risk of pancreatitis. Abbreviations: eHTG, extreme hypertriglyceridemia (TG ≥1000 mg/dL); FA, fatty acid; FCS, familial chylomicronemia syndrome; LPL, lipoprotein lipase; MCS, multifactorial chylomicronemia syndrome; PC, persistent chylomicronemia; TG, triglycerides. ^*^ Three measurements ≥1000 mg/dL over 6 to 12 weeks. ^**^ Alarm features: recurrent acute pancreatitis not caused by ethanol or cholelithiasis, recurrent hospitalizations for severe abdominal pain without another identified cause, childhood pancreatitis, family history of hypertriglyceridemia-induced pancreatitis, and/or postheparin LPL activity <20% of normal value. ^***^ TG ≥1000 mg/dL in more than half of measurements without alarm features listed above.

Considering PC as a category encompassing patients with a wide range of risk for complications, we suggest classification of PC into 4 subtypes. These subtypes can help guide tailored management recommendations based on the severity of the phenotype and associated risk of pancreatitis (Fig 3). The first and most severe subtype comprises individuals with genetically documented FCS. The second includes patients with clinical FCS, who also have severe chylomicronemia with a high likelihood of FCS based on the available scoring systems but either have not had genetic testing or lack a classic biallelic monogenic deficit.^18,19,28^ Given the ultrarare prevalence of FCS (both genetic and clinical), most patients with PC will be in subtypes 3 and 4 (collectively, refractory MCS).^15,16,27^ The third subtype is “PC with alarm features” and consists of individuals with chylomicronemia on at least 3 occasions with at least 1 alarm feature: (1) history of recurrent acute pancreatitis not caused by cholelithiasis or ethanol, (2) recurrent hospitalizations for severe abdominal pain without another identified cause, (3) pancreatitis in childhood, (4) family history of hypertriglyceridemia-induced pancreatitis, (5) postheparin LPL activity <20% of normal value (not widely available in the clinical setting).

These features were used for enrollment into a recent clinical trial and identified a subset of individuals with PC who have a high risk of pancreatitis comparable to that in FCS patients^60,61^ (further detail provided in the next section). While subtypes 1 to 3 are considered very high risk, the fourth subtype, “PC with no alarm features,” is still considered high risk and consists of individuals with recurrent TG ≥1000 mg/dL in more than half of multiple measurements after making lifestyle modifications and receiving medical interventions.^11^ As discussed earlier, the risk of pancreatitis in these patients is higher than in patients with isolated or intermittent chylomicronemia but lower than the risk in FCS.^11^ The prevalence of PC using the aforementioned definitions is 1 in 5500 in the United States, which meets the definition of a rare disease (affecting fewer than 200,000 people in the country) according to the Rare Diseases Act of 2002.^62^

## Approach to assessment and treatment of persistent chylomicronemia

Once chylomicronemia (TG ≥1000 mg/dL) is identified, healthcare professionals should identify secondary factors that may contribute to elevated TG levels, such as poorly controlled diabetes, obesity, metabolic syndrome, ethanol intake, suboptimal dietary habits, inactivity, and TG-raising medications (see Table 2).^13^

### Lifestyle measures

Briefly, lifestyle modification should be initiated as a foundational approach to manage eHTG (chylomicronemia), including a diet very low in fat (<10%–15% daily caloric intake), reduction in simple/refined carbohydrates, avoidance of high fructose intake, avoidance of ethanol intake, exercising regularly (while not specific to these patients, ≥150 minutes of moderate-intensity physical activity or ≥75 minutes of vigorous-intensity physical activity is recommended per week for overall cardiovascular health),^63^ and regulating and maintaining a healthy weight in patients with overweight or obesity.^13^ Referral to a dietitian expert in low-fat diet should be considered. The role of lifestyle modification and details on the consumption of essential fatty acids and use of medium-chain TG to allow a small amount of fat intake without aggravating chylomicronemia have been reviewed elsewhere.^13^

### Conventional triglyceride-lowering medications

Together with lifestyle interventions, first-line TG-lowering agents, including fibrates and marine omega-3 fatty acids, should be prescribed and their effectiveness evaluated.^64^ Fibrates are modulators of peroxisome proliferator–activated receptor *α* (PPAR*α*), and their effect is mainly related to LPL upregulation, resulting in minimal TG lowering in individuals with no LPL activity.^65^ Prescription marine omega-3 fatty acids (eicosapentaenoic acid and docosahexaenoic acid, not linolenic acid) reduce TG synthesis in the liver, resulting in reduced secretion of VLDL, and have a small effect on LPL upregulation and chylomicron clearance in the exogenous pathway.^64,66,67^ Any omega-3 intake (eg, 2 g twice daily) must be considered as part of the individual’s daily fat limit.^16,68^ Both these medication classes are more effective when endogenous pathways and VLDL accumulation contribute significantly to elevated TG and LPL activity is present.^13,69,70^ Statins are indicated to reduce any excess cardiovascular risk and may modestly lower TG levels in patients who do not have FCS.^4^ Orlistat (an intestinal lipase inhibitor), which reduces intestinal fat absorption and thus chylomicron TG content by about one-third,^71,72^ and niacin, which reduces hepatic TG secretion,^73^ may also be considered. Although glucagon-like peptide 1 (GLP1) receptor agonists have not traditionally been considered TG-lowering medications, they are increasingly utilized in clinical practice for patients with diabetes and/or obesity, given their beneficial effects on TG reduction. Despite cautionary labels regarding the potential risk of pancreatitis, more recent data from large placebo-controlled randomized trials of treatment with GLP1 receptor agonists have not demonstrated an increased risk of pancreatitis compared with placebo.^74–76^

### Referral to a lipid expert and multidisciplinary lipid clinic

If eHTG (chylomicronemia) persists after addressing secondary causes, implementing lifestyle modifications, and trying fibrate therapy and possibly 1 or more of the other TG-lowering agents, a diagnosis of PC should be considered. In such cases, the patient may benefit from referral to an expert in lipidology for further evaluation and treatment. Assessment of available serial TG measurements to document the frequency and severity of chylomicronemia episodes is needed to establish the presence of PC and its subclassification. Assessing the risk of pancreatitis, particularly in individuals with a history of pancreatitis, is crucial to determine the necessity for additional therapeutic interventions beyond conventional medications.^45,46^ The presence of “alarm” features (particularly prior history of acute pancreatitis) should be evaluated to estimate the risk of future pancreatitis. Genetic testing may be performed to characterize the etiology and subtype of PC, and FCS scoring algorithms can be used to determine the likelihood of clinical FCS.

### Beyond conventional treatment: Apolipoprotein C-III inhibitors

ApoC-III is a glycoprotein that inhibits LPL and hepatic lipase activity and interacts with apolipoprotein E (apoE). Inhibition or decreased production of apoC-III can dramatically reduce TG through both LPL-dependent and independent mechanisms, including increased hepatic clearance of TRL, reduced VLDL secretion, and reduced intestinal lipid absorption of dietary TG.^77,78^ Two medications in this class are olezarsen (an antisense oligonucleotide targeting apoC-III) and plozasiran (a small interfering RNA [siRNA] targeting apoC-III).

Two pivotal trials investigated the effects of olezarsen and plozasiran on TG lowering and pancreatitis risk in adult patients with chylomicronemia. In the Balance trial, 66 “genetic FCS” patients were randomized to receive either placebo or olezarsen. Notably, olezarsen markedly reduced mean TG levels, −43% (95% CI, −69 to −18; *P* < .001) in the 80 mg monthly arm and −22% (−47 to 3; *P* = .08, not statistically significant) in the 50 mg monthly arm, and reduced the risk of pancreatitis (a secondary endpoint) by 88% (rate ratio 0.12; 95% CI, 0.02 to 0.66).^60^ The PALISADE trial investigated the ability of plozasiran to reduce TG and pancreatitis risk in patients with chylomicronemia. In this trial, patients were initially enrolled based on a genetic diagnosis of biallelic FCS, but subsequently, at the suggestion of regulatory authorities, the inclusion criteria were broadened to include patients with very-high-risk PC defined as a documented history of fasting TG levels >1000 mg/dL on at least 3 occasions with “alarm” features that included (1) history of recurrent episodes of acute pancreatitis; (2) recurrent hospitalizations for severe abdominal pain; (3) childhood pancreatitis; (4) family history of hypertriglyceridemia-induced pancreatitis; (5) positive genetic testing or postheparin LPL activity <20%. Accordingly, 75 patients were included, 58% with FCS and 42% with PC and “alarm” features but with-out biallelic pathogenic variants in genes causative of FCS. In this trial, plozasiran led to reductions in median TG levels, −80% (−90 to −61) in the 25 mg every 3 months arm and −78% (−88 to −49) in the 50 mg every 3 months arm, and reduced pancreatitis risk by 83% (odds ratio 0.17; 95% CI, 0.03 to 0.94) after 10 months of treatment. The observed benefits were irrespective of genotype (ie, FCS vs non-FCS).^61^

The clinical criteria in the latter phase of recruitment for PALISADE were useful to identify a subset of individuals with PC who had a very high frequency of prior pancreatitis (89% of the study population) and also during the 1-year duration of the clinical trial (20% incidence in the placebo arm).^61^ These frequencies are comparable to those among genetically confirmed FCS patients in the Balance trial (71% and 30%, respectively).^60^ Therefore, patients enrolled in these trials represented the most severe PC cases and highlighted the importance of treatment with apoC-III inhibitors to minimize the risk of acute pancreatitis and its sequelae.

In the clinical setting, TG should be measured after implementation of lifestyle management and first-line pharmacotherapy, and individuals with 3 measurements ≥1000 mg/dL over 6 to 12 weeks, as well as any of the alarm features, meet the criteria for PC and should be considered at very high risk for complications. Although no trial has been specifically designed for patients with “clinical FCS,” particularly with “alarm” features, the PALISADE trial can be regarded as the most relevant source of information about effects of treatment with anti–apoC-III therapies in these patients (see Fig 3).

Olezarsen was approved by the US Food and Drug Administration (FDA) to reduce TG in patients with FCS in December 2024, and the data reviewed here and elsewhere suggest that patients with clinical FCS and PC with “alarm” features may also benefit from treatment with this agent. The New Drug Application for plozasiran for the same indication was accepted in January 2025, and the Prescription Drug User Fee Act review deadline is in November 2025. Both medications are currently being studied for a broader spectrum of patients with TG elevation, including sHTG, intermittent chylomicronemia, and less severe forms of PC.^61,79–81^ Data are lacking on the use of these medications in nonadult patients with these conditions.

### Management of patients with persistent chylomicronemia and no alarm features: Subtype 4

PC patients without “alarm” features, classified as PC subtype 4, have lower risk of pancreatitis compared to subtypes 1 to 3, but have high risk of pancreatitis, particularly patients with a single prior episode of pancreatitis. Many patients with chylomicronemia are not effectively treated, allowing its persistence and elevated risk of pancreatitis. Identifying patients with PC subtype 4 with no “alarm” features who are at high risk of pancreatitis can provide an opportunity for earlier intervention that may improve outcomes. Although no data from clinical trials are available to guide individualized treatment in this group, the observational data described above demonstrated a high occurrence (26%) of potentially preventable TG-induced pancreatitis in patients with PC subtype 4.^11^ Therefore, these patients need to be offered intensive lifestyle modifications, treatment of causes of secondary hypertriglyceridemia, treatment with conventional TG-lowering medications including combination therapy if needed, and frequent TG monitoring, as well as patient education about the risks and symptoms and signs of pancreatitis.^82^ Efforts should be made to identify individuals at greatest risk of pancreatitis within this group (eg, those with a history of pancreatitis, positive genetic testing for heterozygous pathogenic variants in genes involved in TG metabolism or high polygenic score for TG, or TG >2000 mg/dL)^26,44^ and to consider additional treatment in these patients. Additionally, abdominal pain episodes in these patients should be carefully evaluated for pancreatitis. This can be done by measuring TG levels at the time of abdominal pain and plasma amylase and/or lipase levels and imaging the pancreas (using ultrasound and/or computed tomography) as clinically indicated. Recurrent episodes of pancreatitis or abdominal pain with no other etiology will reclassify these patients to higher risk categories (ie, PC with alarm features). Ultimately, patient–clinician discussions should involve shared decision-making to explore the possible use of newer treatment options for managing PC, recognizing the limitations of approval criteria from the FDA and other regulatory agencies (see Fig 3).

### Multidisciplinary care

Optimal care for all patients with PC necessitates the collaboration of a multidisciplinary team, including lipidologists, dietitians, primary care health professionals, diabetologists, pancreatologists (especially in patients with a history of recurrent pancreatitis), and cardiologists if there is elevated risk of ASCVD. Additionally, psychologists, counselors, nurses, social workers, pharmacists, and patient support groups are helpful for personalized care, addressing social factors contributing to and resulting from PC, and improving health-related quality of life. Healthcare professionals in private or small group practices may have difficulty accessing this necessary team of providers, but referral to specialized clinics may help overcome barriers to team-based care. Women with PC need special advice concerning contraception and management during pregnancy in collaboration with their gynecologist or obstetrician and primary care clinician; aggravation of chylomicronemia with hormonal contraception and during pregnancy is associated with increased risk of pancreatitis.

### Other emerging therapies

#### ANGPTL3 inhibitors.

Evinacumab (a monoclonal antibody), zodasiran (siRNA), and solbinsiran (siRNA) are ANGPTL3 inhibitors that can treat a large spectrum of lipid disorders, from severe refractory hypercholesterolemia to chylomicronemia.^83,84^ However, their effect on TG reduction is LPL-dependent, and patients with no or very low LPL activity, as in FCS, do not achieve significant TG lowering in response to treatment with ANGPTL3 inhibitors, despite substantial decreases in circulating apoC-III.^83–86^ The efficacy of these agents has been assessed in patients with mixed dyslipidemia, mHTG, sHTG, and eHTG, demonstrating up to 82% TG reductions, but none are currently in ongoing development for the treatment of chylomicronemia. Evinacumab is FDA-approved for LDL-C lowering only in patients with homozygous FH.^83,85,87–89^ Other ANGPTL3, ANGPTL3/8, or ANGPTL4 inhibitors are in development and might be useful in subgroups of patients with PC having residual LPL bioavailability.

#### FGF21 analogs.

Fibroblast growth factor 21 (FGF21) is a peptide hormone secreted by the liver, adipose tissue, skeletal muscles, and pancreas that affects energy expenditure and metabolism. Pegozafermin is an FGF21 analog, for which initial studies showed ~60% TG reduction across a wide range of hypertriglyceridemia, as well as multiple other metabolic benefits, including improved markers of insulin resistance, weight loss, and improvement of hepatic steatosis.^90–92^ An ongoing phase 3 trial of pegozafermin is enrolling patients with sHTG without genetically proven FCS (NCT05852431).

## Summary and conclusions

Until very recently, there were no effective treatments for patients with severe forms of chylomicronemia besides lifestyle modifications and conventional TG-lowering medications, and these generally had extremely limited efficacy. The current diagnostic approach for chylomicronemia is focused on genotypic categorization of patients into FCS and MCS, but an update in the approach is needed. Nevertheless, our current understanding of chylomicronemia management now extends beyond purely genetic classifications, emphasizing phenotype severity and associated risks to guide more precise therapeutic interventions. We have described in detail the rationale for proposing the term PC, defined as TG above 1000 mg/dL in more than half of the measurements, which identifies adult patients with chylomicronemia with a high disease burden and high risk of pancreatitis. While further research is needed to refine and better characterize the definition of PC, the current proposal is a template for transitioning the classification of chylomicronemia from genetic criteria to more pragmatic and clinically focused criteria. We introduced “alarm” features, which include (1) history of recurrent TG-induced acute pancreatitis, (2) recurrent hospitalizations for severe abdominal pain without another identified cause, (3) childhood pancreatitis, (4) family history of TG-induced pancreatitis, and/or (5) postheparin LPL activity <20% of normal value, which can identify PC cases with very high risk of pancreatitis comparable to the risk in patients with FCS. The recent clinical availability of the first apoC-III inhibitor, olezarsen, has greatly improved our ability to treat PC, with a second agent potentially becoming available in late 2025. Although the current FDA approval of olezarsen is for treatment of patients with FCS, expanded indications beyond the current approval criteria should be further evaluated for very-high-risk PC such as individuals with alarm features.

## Future direction

Considering that pancreatitis is the most severe complication of chylomicronemia, further research is warranted to identify the clinical, environmental, and host factors, including genetic predisposition and imaging characteristics, associated with increased risk of pancreatitis. These findings should be derived from large, diverse populations worldwide, encompassing various ethnic, racial, and socioeconomic backgrounds. By adopting this personalized approach, we can define treatment thresholds for each patient based on individual characteristics and risk profile. Our proposed approach, which aligns with the general principles for improving the quality of healthcare, provides a strategy to make available safe, effective, patient-centered, timely, efficient, and equitable care for all patients with PC.^93^

## Figures and Tables

**Table 1:** Current classification of chylomicronemia.^12, 15, 18, 23–25^

	FCS	MCS

Population frequency	1–10:1,000,000	1:600 to 1:250
Fasting TG, mg/dL	≥1000	≥1000
Primarily elevated lipoprotein fractions	Chylomicrons	Chylomicrons and chylomicron remnants and VLDL and IDL
Genetic basis	Biallelic monogenic (recessive)	Most often polygenic
Relevant genetic determinants	Biallelic combination of pathogenic variants in LPL or genes encoding proteins required for *LPL* function (*APOC2, GPIHPB1, APOAV, LMF1*)	Susceptibility may be due to rare variants in genes canonically or peripherally involved in TG metabolism, including heterozygous pathogenic variants in LPL machinery, and/or the accumulation of common, small-effect TG-raising SNPs
Role of nongenetic factors	May modulate the severity of phenotype, but not its presence	Typically modulates the presence and severity of phenotype
Age at presentation	Most often presents in childhood	Most often presents in adulthood
ApoB-100/LDL-C	Low	Can be elevated
Risk of ASCVD	Low	Variable, can be high compared to general population
BMI	Most often normal (can be low)	Patients are often overweight or obese
Risk of acute pancreatitis	Very high, generally higher than in MCS	Increased, but generally lower than in FCS
Chylomicronemia course	Persistent	Can be persistent or intermittent
Response to fibrates or omega-3	Very little or none	Modest to substantial

Abbreviations: ApoB-100, apolipoprotein B-100; ASCVD, atherosclerotic cardiovascular disease; BMI, body mass index; FCS, familial chylomicronemia syndrome; IDL, intermediate-density lipoprotein; LPL, lipoprotein lipase; LDL-C, low-density lipoprotein cholesterol; MCS, multifactorial chylomicronemia syndrome; omega-3, marine omega-3 fatty acids (eicosapentaenoic acid [EPA] and docosahexaenoic acid [DHA]); SNP, single nucleotide polymorphism; TG, triglycerides; VLDL, very-low-density lipoprotein.

**Table 2:** Causes or triggers of intermittent or persistent chylomicronemia beyond genetic defects in the LPL machinery.^4, 30–34^

Categories	Examples

Hormonal changes	Pregnancy, poorly controlled diabetes, central obesity with insulin resistance and metabolic syndrome, hypothyroidism, acromegaly, Cushing syndrome
Lifestyle factors	Ethanol consumption, high-fat diet, carbohydrate-rich diet, high fructose intake, sedentary lifestyle, cannabis use
Immunological diseases	Rheumatoid arthritis, psoriasis, systemic lupus erythematosus, Weber–Christian disease
Hematological malignancies	Multiple myeloma, lymphoproliferative disorders, paraproteinemia
Renal diseases	Nephrotic syndrome, chronic kidney disease
Medications	Oral estrogens, tamoxifen, corticosteroids, androgens, retinoids and retinoid X receptor agonists, some immunosuppressants, anti-HIV agents (protease inhibitors), thiazides, beta blockers, second-generation antipsychotics, SSRIs, valproic acid (Depakene), propofol, bile-acid sequestrants
Other genetic factors	Primary partial lipodystrophy, primary generalized lipodystrophy, elevated HTG polygenic scores, dysbetalipoproteinemia (type III; intermittent chylomicronemia may occur), glycerol kinase deficiency (pseudo-HTG), glycogen storage disease (type 1a, G6PC1 mutation)
Other conditions	Myotonic dystrophy, amyloidosis, HIV infection, secondary lipodystrophy (eg, due to antiretroviral therapy for HIV), circulating antibodies against LPL or GPIHBP1, epigenetic factors (DNA or histone methylation)

Abbreviations: G6PC1, glucose-6-phosphatase catalytic subunit 1; GPIHBP1, glycosylphosphatidylinositol-anchored high-density lipoprotein binding protein-1; HbA1C, hemoglobin A1C; HIV, human immunodeficiency virus; HTG, hypertriglyceridemia; LPL, lipoprotein lipase; SSRIs, selective serotonin reuptake inhibitors.

## Purpose of statement

In this consensus statement, we briefly review the metabolism of triglyceride-rich lipoproteins (TRL), the epidemiology of chylomicronemia, and clinical and diagnostic differences between familial chylomicronemia syndrome (FCS) and multifactorial chylomicronemia syndrome (MCS). We also provide the rationale for a new pragmatic diagnostic approach and a paradigm shift in defining adults with persistent chylomicronemia (PC) along with alarm features for risk stratification and recommendations on management options. This Expert Clinical Consensus document was endorsed by the National Lipid Association (NLA) and the American Society for Preventive Cardiology (ASPC).
